# Engineered Graphene Material Improves the Performance of Intraneural Peripheral Nerve Electrodes

**DOI:** 10.1002/advs.202308689

**Published:** 2024-06-11

**Authors:** Bruno Rodríguez‐Meana, Jaume del Valle, Damià Viana, Steven T. Walston, Nicola Ria, Eduard Masvidal‐Codina, Jose A. Garrido, Xavier Navarro

**Affiliations:** ^1^ Institute of Neurosciences Department of Cell Biology Physiology and Immunology Universitat Autònoma de Barcelona Bellaterra 08193 Spain; ^2^ Centro de Investigación Biomédica en Red en Enfermedades Neurodegenerativas (CIBERNED) Instituto de Salud Carlos III Madrid 28031 Spain; ^3^ Department de Bioquímica i Fisiologia Universitat de Barcelona Barcelona 08028 Spain; ^4^ Catalan Institute of Nanoscience and Nanotechnology (ICN2) CSIC and BIST Campus UAB Bellaterra 08193 Spain; ^5^ ICREA Barcelona 08010 Spain; ^6^ Institut Guttmann of Neurorehabilitation Badalona 08916 Spain

**Keywords:** foreign body reaction, graphene, intraneural electrode, neuroprostheses, recording, stimulation

## Abstract

Limb neuroprostheses aim to restore motor and sensory functions in amputated or severely nerve‐injured patients. These devices use neural interfaces to record and stimulate nerve action potentials, creating a bidirectional connection with the nervous system. Most neural interfaces are based on standard metal microelectrodes. In this work, a new generation of neural interfaces which replaces metals with engineered graphene, called EGNITE, is tested. In vitro and in vivo experiments are conducted to assess EGNITE biocompatibility. In vitro tests show that EGNITE does not impact cell viability. In vivo, no significant functional decrease or harmful effects are observed. Furthermore, the foreign body reaction to the intraneural implant is similar compared to other materials previously used in neural interfaces. Regarding functionality, EGNITE devices are able to stimulate nerve fascicles, during two months of implant, producing selective muscle activation with about three times less current compared to larger microelectrodes of standard materials. CNAP elicited by electrical stimuli and ENG evoked by mechanical stimuli are recorded with high resolution but are more affected by decreased functionality over time. This work constitutes further proof that graphene‐derived materials, and specifically EGNITE, is a promising conductive material of neural electrodes for advanced neuroprostheses.

## Graphical abstract

EGNITE is a newly graphene‐based material developed for interfacing the nervous system. Histological and functional analyses revealed that the EGNITE integrated properly into the peripheral nerve. EGNITE stimulation and recording capabilities overperformed those of other conductive materials commonly used in neural electrodes.

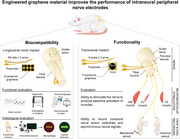

## 1 Introduction

Loss of sensory and/or motor function as a result of nerve injury (e.g., spinal cord injury, brachial plexus injury) or loss of a limb (e.g., amputation) affects several million people worldwide, serving as a powerful motivation for the development of new rehabilitation strategies. Limb neuroprostheses based on interfacing the peripheral nervous system (PNS) are designed in a way that neural electrodes record motor signals from the patient residual efferent nerves to control the motion of the bionic limb, whereas other electrodes provide sensory feedback collected from artificial sensors in the bionic limb by stimulating the afferent nerve fibers, thus, constituting a bidirectional interface with the nervous system.^[^ ^1^ ^]^ In the last decades, a variety of such peripheral nerve interfaces have been developed and tested.^[^ ^2^, ^3^ ^]^ However, translation of these research efforts into clinical applications is rather slow due to technological challenges. In the case of nerve interfaces, the main challenges are 1) recording of action potentials traveling along the nerves with high signal‐to‐noise ratio (SNR) and from multiple targets, with high spatial resolution, 2) highly selective stimulation of small populations of nerve fibers, and 3) low foreign body reaction (FBR) for chronic applications.

In neuroprosthetic applications an electrical coupling is commonly used to interconnect the nervous system with the prosthetic devices. Currently, most nerve interfaces are based on metal, such as platinum, gold or iridium, microelectrodes fabricated onto flexible substrates.^[^ ^2^ ^]^ However, inherent limitations such as stiffness, low biocompatibility, limited charge injection when reduced to a micrometric scale or limited stability to stimulate safely over extended periods, have motivated the exploration of non‐metallic alternatives. Non‐metal electrodes have emerged as promising candidates, offering advantages in terms of flexibility, reduced tissue injury, and enhanced signal resolution. Such electrodes are based on material substrates with either an organic composition or a modified metallic structure.^[^ ^4^ ^]^ Conductive polymers such as polyacetylene, polypyrrol or poli(3,4‐etilendioxitiofeno) (PEDOT), achieve high conductivities via doping. These conjugated polymers have shown capability for neural stimulation, good biocompatibility, and integration with nerve cells.^[^ ^5^, ^6^, ^7^ ^]^ Carbon‐based electrodes are another promising alternative to serve as effective recording and stimulation devices. In some cases, carbon nanomaterials have been introduced as dopants or coatings to improve the conductivity of silk fibers or conducting polymer electrodes.^[^ ^8^ ^]^ However, coating materials may compromise the long‐term stability of the electrodes and restrict available sterilization methods due to compatibility issues.^[^ ^9^ ^]^ In other developments, carbon nanotube thin filaments were used to record neural activity from neuronal ensembles,^[^ ^10^ ^]^ and highly flexible carbon nanotube yarns produced reliable and stable chronic recordings in the peripheral nerves, outperforming current metal‐based electrodes in recording quality, stability, and selectivity.^[^ ^11^ ^]^ However, in carbon fiber electrodes, the number of contacts is limited to one per electrode, in contrast with state‐of‐the‐art intraneural electrodes, such as tfLIFE and TIME,^[^ ^12^, ^13^ ^]^ that allow to place multiple active contacts per device. A new generation of neural interfaces based on carbon nanotubes and graphene are increasingly used for micro‐scale electrode fabrication, due to their superior electrical properties.^[^ ^14^ ^]^ However, limited electrochemical performance of single‐layer graphene microelectrodes restricts the scope for downsizing.^[^ ^15^ ^]^ Multilayer porous electrodes have been explored to improve performance.^[^ ^16^ ^]^ In a recent work, a reduced‐graphene oxide material, named Engineered Graphene for Neural Interface (EGNITE), was used for the micromachining fabrication process of flexible microelectrode arrays on polyimide (PI) as substrate for high spatial resolution of neural recording and stimulation in the central and peripheral nervous system, overcoming the challenges associated with microelectronic processing of high charge injection thin‐films.^[^ ^17^ ^]^

For biomedical applications, it is important that the capability of the device as a bidirectional interface is maintained over extended periods of time. Once implanted, a neural interface must remain within the body of the subject for months or years, so the stability of the materials in the electrode is crucial. Time‐dependent loss of neuron‐device communication limits the long‐term use of such devices. Decreasing functionality is associated with reactive responses that produce an encapsulating cellular reaction around electrodes implanted in the nervous system. Therefore, a major prerequisite for the application of novel nerve electrodes is that the implant must be biocompatible, and also that the organism embodies the interface without creating a thick insulating capsule. After insertion of a device in the peripheral nerve early and chronic cellular responses occur in a subset of processes known as FBR.^[^ ^12^, ^18^, ^19^, ^20^, ^21^ ^]^ The acute phase of inflammation is primarily triggered by the traumatic insertion of the electrode that induces blood vessels damage, disrupts extracellular matrix and neuronal processes, and recruits macrophages. The chronic response is mainly characterized by the recruited macrophages that contribute both to debris elimination and inflammatory response in the nerve, and also the activation of fibroblasts that form a healing scar formed by collagen and other extracellular proteins.

In this work, we have assessed whether novel multichannel microelectrodes, based on EGNITE^[^ ^17^ ^]^ deposited on PI are suitable for peripheral nerve interfacing. To this end, in vitro and in vivo biocompatibility and in vivo functionality of EGNITE material were assessed. To assess in vitro biocompatibility cortical and ganglion cells were seeded on top of EGNITE. To evaluate in vivo integration, EGNITE devices were implanted in the sciatic nerve of rats for chronic evaluation of functional and morphological changes in the nerve along three months. To assess in vivo functionality EGNITE devices were implanted in the sciatic nerve of rats for nerve stimulation and recording experiments.

## 2 Results

### 2.1 Biocompatibility Study

#### 2.1.1 EGNITE Does Not Decrease Neuronal Viability

**Figure 1:**
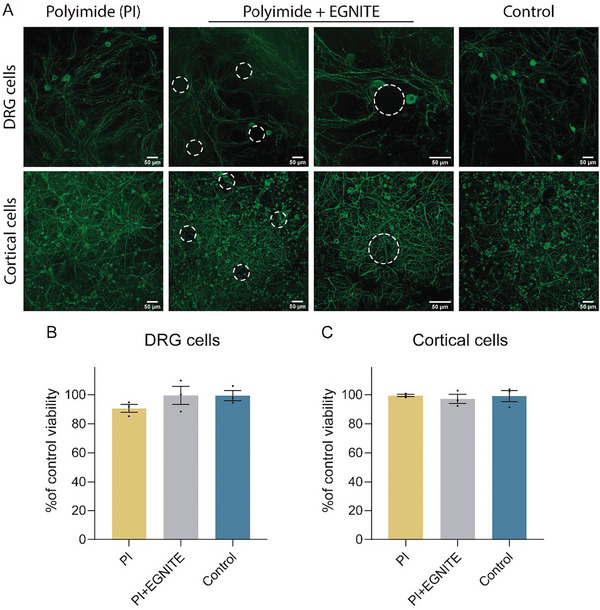
In vitro biocompatibility assays of EGNITE. A) DRG neurons (top micrographs) and cortical neurons (bottom micrographs), labeled in green by immunolabeling β3 Tubulin after 4 and 7 days in culture, respectively, on top of control glass, PI and PI containing EGNITE. Scale bar: 50 µm. B,C) Histogram plots of neuronal viability assessed by the MTT test after 4 (DRG, n = 3) and 7 (cortical neurons, n = 3) days in vitro, without significant differences between the three surfaces. Data are represented as mean ± SEM. *p* > 0.05, one‐way ANOVA followed by Dunnet's multiple comparison test.

Both dorsal root ganglion (DRG) and cortical neurons grew well on top of the PI test samples with and without EGNITE (**Figure** **1**A). In the case of cortical cell culture, EGNITE dots were not even visible, as the device surface was completely covered by a layer of cells. The results of the MTT test showed that neuronal survival of both ganglion (Figure 1B) and cortical neurons (Figure 1C) was similar in the three conditions tested proving that both PI and EGNITE are not toxic in vitro.

#### 2.1.2 EGNITE Implants In Vivo Do Not Affect Nerve Function

**Figure 2:**
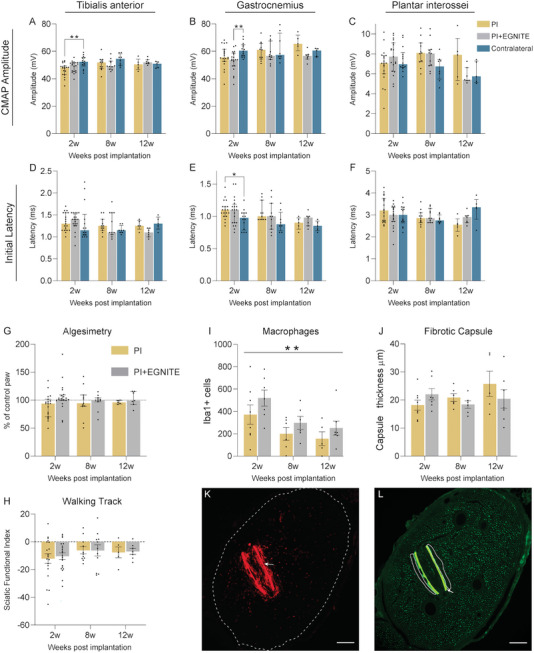
In vivo results show that EGNITE‐containing devices are biocompatible. A–F) Motor nerve conduction parameters of animals implanted with PI or PI + EGNITE devices and the control contralateral paws for 12 weeks follow‐up. (A–C) CMAP amplitude of TA (A), GM (B) and plantar interosseus (PL) (C) muscles. D–F) CMAP onset latency of TA (D), GM (E), and PL (F) muscles. ^*^ *p* < 0.05 ^**^ *p* < 0.01 Kruskal–Wallis followed by Dunn's multiple comparison test for each time point. Data are represented as median and interquartile range. G) Algesimetry test results expressed as percentage of force withdrawal (versus contralateral control paw) of animals implanted with PI and PI with EGNITE devices; *p* > 0.05 two‐tailed Mann–Whitney for each time point. Data are represented as median and interquartile range. H) Plot of the SFI obtained in the walking track test; *p* > 0.05 two‐way ANOVA followed by Sidak's multiple comparison test. Data are represented as mean ± SEM. For functional tests, at 2w PI n = 19, PI+EGNITE n = 20, Contralateral n = 14; at 8w PI n = 11, PI+EGNITE n = 11, Contralateral n = 8; at 12w PI n = 5, PI+EGNITE n = 7, Contralateral n = 5. I) Number of inflammatory Iba1+ cells in the tibial nerve of animals implanted with PI devices with and without EGNITE (2w PI n = 8, PI+EGNITE n = 7; 8w PI n = 6, PI+EGNITE n = 6; 12w PI n = 5, PI+EGNITE n = 7). ^**^ *p* < 0.01 versus time, two‐way ANOVA followed by Sidak's multiple comparison test. Data are represented as mean ± SEM. J) Tissue capsule thickness around the devices in the tibial nerve of animals implanted with PI devices with and without EGNITE (2w PI n = 8, PI+EGNITE n = 7; 8w PI n = 6, PI+EGNITE n = 6; 12w PI n = 5, PI+EGNITE n = 7). *p* > 0.05, two‐way ANOVA followed by Sidak's multiple comparison test. Data are represented as mean ± SEM. K,L) Representative images of a tibial nerve labeled for inflammatory cells (Iba1, K) or axons (RT97, L) implanted with a device (arrow points to the autofluorescent PI strips) at 2 weeks post implantation in the tibial fascicle (dashed line in K) and the tissue capsule (dotted line in L). Scale bar: 100 µm.

After 8 and 12 weeks of follow‐up post implantation in the sciatic nerve there were no significant changes in the electrophysiological results of the three groups of rats (control/contralateral, PI and PI with EGNITE). Only a reduction in the amplitude of the tibialis anterior (TA) compound muscle action potential (CMAP) in the PI group and the GM CMAP in the PI + EGNITE group at 2 weeks (**Figure** **2**A–C) was found; since the TA muscle is innervated by the peroneal fascicle, where the implants were not placed, and the amplitudes of both muscles recovered at 8 weeks, these decrease can be attributed to the surgery alone and not to the implant. The latency of the gastrocnemius medialis (GM) CMAPs showed a slight increase in the PI group at 2 weeks. From 8 weeks onward, the latency of CMAPs did not show differences between groups (Figure 2D–F), indicating no myelin damage.

The algesimetry tests yielded similar values of pain withdrawal threshold between the implanted and contralateral hindlimbs in the two groups of rats (Figure 2G), without evidence of hyperalgesia that might had been induced by nerve compression or injury. In addition, walking track measurements (Figure 2H) did not show significant changes between the hindlimb with the implanted devices and the control contralateral hindlimb during follow‐up. The Sciatic Functional Index (SFI) values were close to zero (normal value) at the different time points. In conclusion, there was no evidence of alterations in the motor and sensory functions conveyed by the sciatic nerve after implanting PI devices containing or not EGNITE.

#### 2.1.3 EGNITE Does Not Exacerbate the Inflammatory Response Compared to the Polyimide Substrate Alone

**Figure 3:**
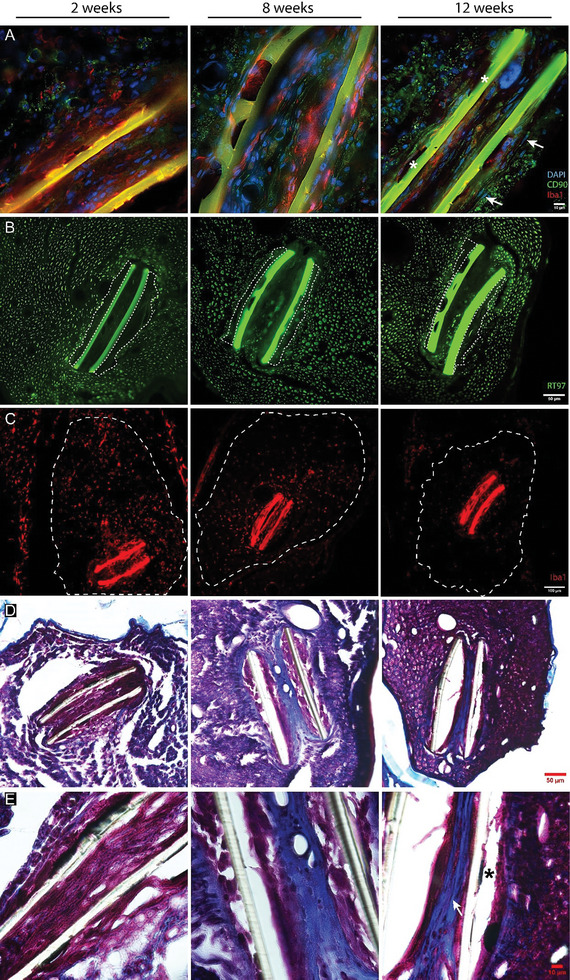
FBR to the EGNITE intraneural implants. Representative micrographs showing the evolution of the inflammatory response over 12 weeks. A) Evolution of the capsule composition, by colabeling macrophages (red Iba 1+), fibroblasts (green CD90+) and cell nuclei (blue DAPI). White arrows point to fusiform cells corresponding to fibroblasts. Asterisks indicate EGNITE dots on top of the PI device. Scale bar: 10 µm. B) Capsule thickness, measured considering the area limited by the dotted lines. Scale bar: 50 µm. C) Infiltrating macrophages (red, Iba 1+ cells) in the area limited by the dotted line that corresponds to the implanted tibial fascicle of the sciatic nerve. Scale bar: 100 µm. D,E) Masson's Trichrome Stain. Capsule composition shifts from densely cellular (pink or purple stain) at 2 weeks to less cellular at 8 and 12 weeks. Note how the acellular component of the capsule (collagen, blue stain) surrounds the PI device at 8 and 12 weeks. Asterisk indicates EGNITE dot on top of the PI device. White arrow points to fusiform cells. Scale bar in (D): 50 µm, in (E): 10 µm.

One of the main events during the FBR is the infiltration by hematogenous macrophages into the implanted tissue, as part of the inflammatory phase. Comparison between implants with and without EGNITE revealed no differences in the number of macrophages present in the tibial nerve (Figure 2I,K). On the other hand, the last phase of the FBR and one of the main problems for the long‐term functionality of intraneural electrodes is the migration of activated fibroblast that secrete collagen and form a dense fibrous capsule around the implant.^[^ ^20^ ^]^ Figure 2J shows that the measured capsule thickness was similar for implants with and without EGNITE at both 2, 8, and 12 weeks, indicating that the presence of EGNITE did not induce damage to the nerve nor further fibrotic scar formation. Immunohistochemical images taken from the implanted nerves (**Figure** **3**B) show numerous axons near the implants at the three time points, indicating limited damage and remodeling after the implant, consistent with previous works.^[^ ^20^, ^22^ ^]^ Regarding the cellular response, macrophages appear as the first cells infiltrating the nerve and surrounding the device at 2 weeks, when the peak of the inflammatory reaction was reported.^[^ ^20^, ^22^ ^]^ By 8 weeks, fibroblasts are present at the edge of the tissue capsule, with a round shape, and acquire a flattened shape by 12 weeks, when the presence of macrophages is markedly reduced. The fibroblasts produce collagen that constitute the capsule covering the PI device (Figure 3D,E). There were no noticeable differences in the microscopic observations between PI devices with and without EGNITE.

Altogether, our in vivo biocompatibility study indicates that EGNITE is suitable for intraneural implantation, since it does not cause any additional damage or neuroinflammatory reaction to that of control PI alone devices.

### 2.2 Functionality Study

During the implantation period rats remained in good health. However, two of the animals damaged the ending part of the device which had been placed under the skin the next day of implantation, so they could not be tested at 30 days post implantation (dpi). No further postoperative complications were observed; the incision wounds healed without inflammatory signs, indicating no gross FBR. Plastic envelopes were found covered by a thin fibrotic tissue, and once removed, revealed the device pads without damage.

#### 2.2.1 EGNITE Microelectrodes Allow Low‐Threshold Current Neuromuscular Stimulation with Good Selectivity

The tests after acute and chronic implantation proved that EGNITE microelectrodes were able to stimulate different axonal subpopulations of the sciatic nerve, depending on the pole used and the applied current intensity.

**Figure 4:**
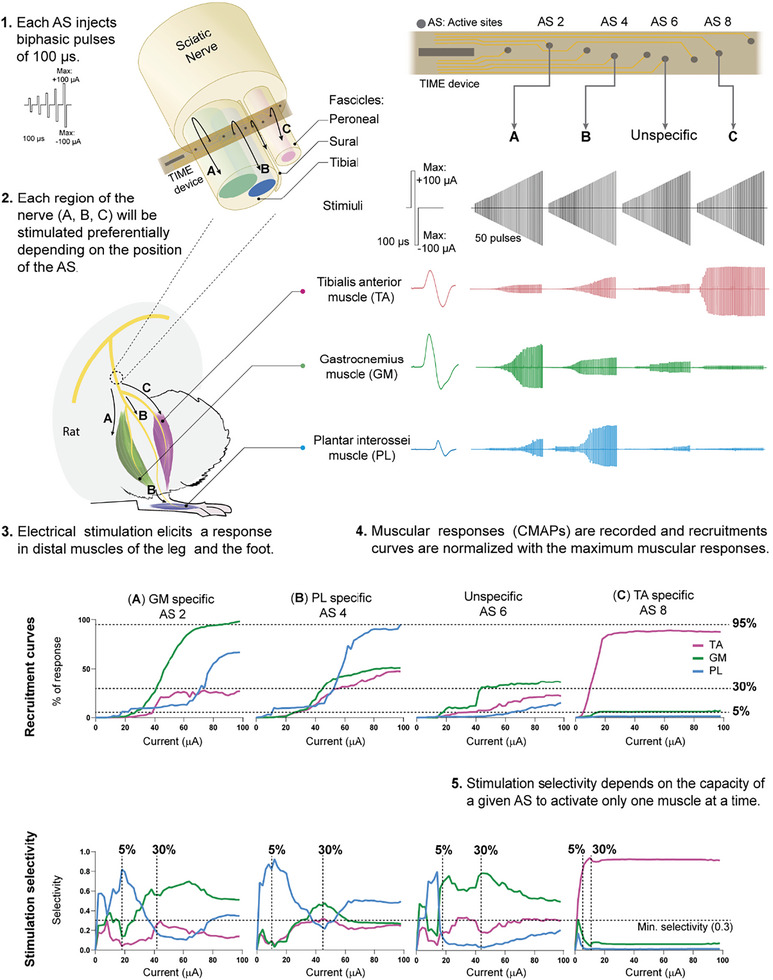
Protocol to assess stimulation capabilities of TIME EGNITE devices. (1) Each electrode injects a sequence of 50 biphasic pulses of increasing current (0–100 µA), 100 µs each at 3 Hz. Each set of 50 pulses is repeated twice. (2) The TIME is inserted transversally into the tibial and peroneal fascicles of the sciatic nerve. Hence, electrodes (AS) interface different regions of the nerve A–C) which innervate different muscles (PL, GM and TA). (3) Electrical stimulation through each of the electrodes elicit a response in distal muscles of the leg and the foot. Muscular responses vary depending on the pole that is used. (4) Monopolar needles are placed in the belly of studied muscles to record the CMAPs produced in each step of the stimulation protocol. CMAPs are normalized to the maximum amplitude for each muscle to build the recruitment curve: a representation of the progressive activation of the muscle as the current increases. The threshold currents needed to activate each muscle at 5%, 30%, and 95% are used for further analyses. (5) Stimulation selectivity measure the capacity of an electrode pole to stimulate one muscle but not the others. Selectivity is calculated at two points: when one of the muscles has reached 5% CMAP amplitude and when a muscle has reached 30% CMAP amplitude. If the three muscles are activated equally the selectivity is minimum (Selectivity index (SI) = 0.33).

**Figure 5:**
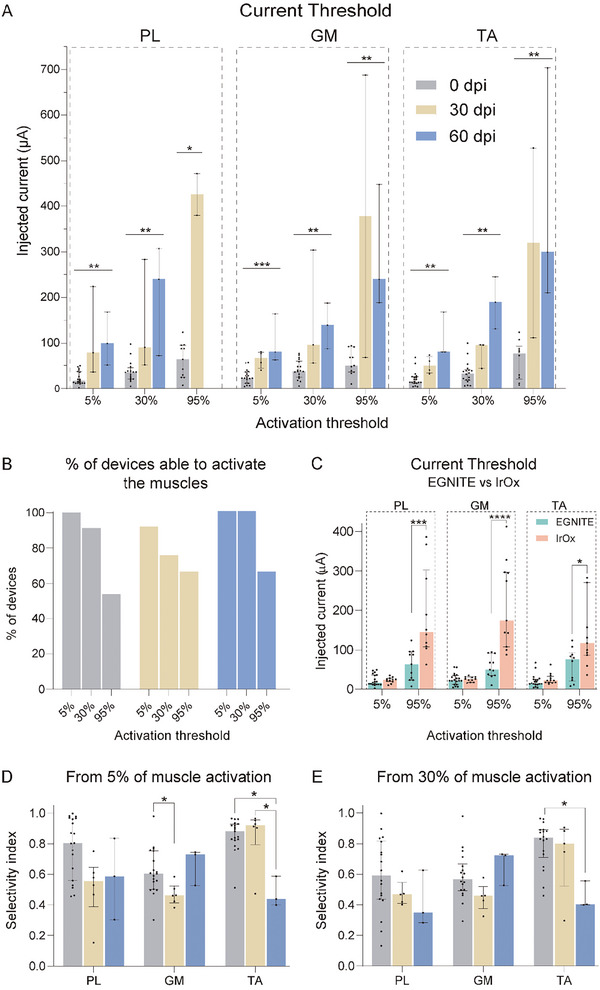
Stimulation capabilities of TIME EGNITE devices in the sciatic nerve. A) Current threshold needed to elicit 5, 30, and 95% of the maximum CMAP amplitude in the PL, GM, and TA muscles over 60 days. PL: 5% 0 dpi n = 19, 30 dpi n = 13, 60 dpi n = 3 ^**^ *p* < 0.01; 30% 0 dpi n = 16, 30 dpi n = 13, 60 dpi n = 3 ^**^ *p* < 0.01; 95% 0 dpi n = 11, 30 dpi n = 2, 60 dpi n = 0 ^*^ *p* < 0.05; GM: 5% 0 dpi n = 19, 30 dpi n = 4, 60 dpi n = 3 ^***^ *p* < 0.001; 30% 0 dpi n = 18, 30 dpi n = 3, 60 dpi n = 3 ^**^ *p* < 0.01; 95% 0 dpi n = 11, 30 dpi n = 2, 60 dpi n = 3 ^**^ *p* < 0.01; TA: 5% 0 dpi n = 19, 30 dpi n = 4, 60 dpi n = 3 ^**^ *p* < 0.01; 30% 0 dpi n = 18, 30 dpi n = 3, 60 dpi n = 3 ^**^ *p* < 0.01; 95% 0 dpi n = 10, 30 dpi n = 2, 60 dpi n = 3 ^**^ *p* < 0.01. Statistical comparisons made with Kruskal–Wallis test followed by Dunn's test for multiple comparison. Data are represented as median and interquartile range. B) Percentage of working devices able to elicit CMAPs of 5, 30, and 95% of the maximum CMAP amplitude in each of the three muscles tested. C) Comparison between TIME electrodes made of EGNITE or iridium oxide (IrOx) of the current needed to elicit 5% and 95% of muscle activation at 0 dpi. IrOx data are extracted from reference.^[^ ^23^ ^]^ PL: EGNITE 5% n = 19 95% n = 11, IrOX 5% n = 11 95% n = 10 ^***^ *p* < 0.001. GM: EGNITE 5% n = 19 95% n = 11, IrOX 5% n = 11 95% n = 11 ^****^ *p* < 0.0001. TA: EGNITE 5% n = 19 95% n = 10, IrOX 5% n = 12 95% n = 11 ^*^ *p* < 0.05, Statistical comparisons made with Mann–Whitney test for each threshold. Data are represented as median and interquartile range. D, E) SI by muscle at 5% and 30% respectively of the maximum CMAP amplitude. PL, GM, TA: 5% 0 dpi n = 19, 30 dpi n = 6, 60 dpi n = 3; PL, GM, TA: 30% 0 dpi n = 18, 30 dpi n = 5, 60 dpi n = 3. ^*^ *p* < 0.05 using Kruskal–Wallis test followed by Dunn's multiple comparison test. Data are represented as median and interquartile range.

Recruitment curves of muscle activity are plotted (**Figure** **4**) for each muscle to calculate the threshold of charge needed to reach 5, 30, and 95% of the maximal CMAP amplitude (**Figure** **5**A). In acute and chronic tests, all EGNITE devices were able to stimulate the nerve up to 5% of the maximal CMAP amplitude. More than 75% of the devices allowed CMAPs higher than 30%, whereas more than half of the devices achieved responses higher than 95% (Figure 5B). The constrain in current delivery of 100 µA, to prevent damage of the EGNITE electrodes, avoided reaching stronger activation of the muscles in some devices. We exceeded this charge injection limit only if the electrode pole did not produce any response at lower levels, particularly in chronic experiments.

Of particular interest is the low current needed to activate the motor nerve fibers (Figure 5A). Compared to previous studies in which TIME devices with 80 µm diameter electrodes of IrOx were used,^[^ ^23^ ^]^ EGNITE electrodes elicited a response with thresholds 2 to 3 times lower (Figure 5C). In chronic tests, current threshold for activation increased (Figure 5A), most probably due to fibrotic encapsulation or electrode deterioration.

For assessing the selectivity to activate each of the three muscles tested, the SI was calculated (Figure 5D,E). The SI for the three tested muscles ranged between 0.6 and 0.9, similar to that found in previous studies using TIME design of electrodes.^[^ ^23^, ^24^ ^]^ Differences in the SI among the set of muscles may be explained due to the different location of axons innervating the muscles inside the sciatic nerve. The SI was calculated when one of the muscles reached 5% and also at 30% of maximal CMAP amplitude. As expected, the selectivity obtained was lower at the latter than at the former level. When a stimulus pulse is given at low intensity from an electrode pole, the current is limited to a small area around and stimulates few axons. The greater the applied current, the greater the stimulated area and, therefore, selectivity is reduced (see Figure 4 bottom graphs). Figure 4 shows an example of an implanted device and how the position of each pole within the sciatic nerve causes the selectivity to shift between the muscles. In this case, pole (or active site, AS) AS8 is placed within the peroneal nerve since it shows high SI for the TA muscle. On the other hand, AS2 and AS4 are located within the tibial nerve, being AS2 likely the closest to the GM fascicle and AS4 in the fascicle supplying the plantar muscles. AS6 is in between the tibial and peroneal nerves and its ability to activate any of the muscles is low.

The SI showed changes during the follow‐up of chronic implants. For the TA muscle there was a drop in the selectivity at 60 dpi. In the PL muscle the selectivity also decreased at 30 dpi. On the contrary GM selectivity remained stable during the follow‐up.

#### 2.2.2 EGNITE Microelectrodes are able to Record Nerve Potentials Induced by Electrical and Mechanical Stimulation

**Figure 6:**
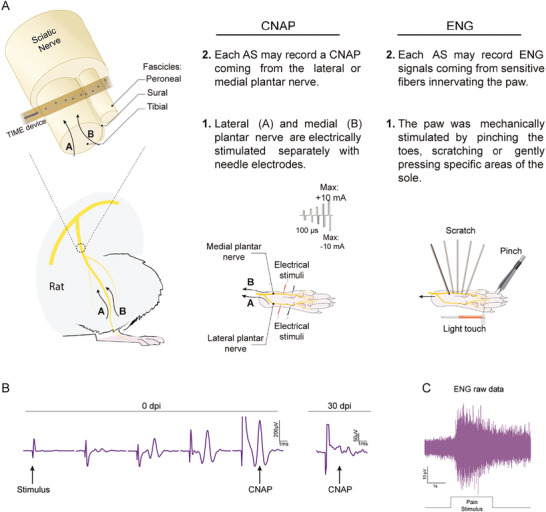
Protocol to analyze recording capabilities of TIME EGNITE devices. A) Recording protocol. The lateral (A) and medial B) plantar nerves were electrically stimulated using monopolar needle electrodes with 50 biphasic pulses of increasing current to a maximum of 10 mA. Elicited CNAPs were recorded though each electrode pole (named AS) in the TIME device. CNAPs are normalized to the maximum valued recorded with hook electrodes around the sciatic nerve. ENG: The paw was mechanically stimulated by pinching the toes with tweezers, scratching the sole with a probe, and pressing specific areas of the sole with a Von Frey filament. Elicited sensory signals were recorded by each pole in the TIME device. (B) Representative recordings of CNAPs elicited by electrical stimulation of the distal plantar nerve at 0 and 30 dpi. C) Representative raw ENG recordings elicited by mechanical noxious stimuli to the paw.

To evaluate the recording capabilities of EGNITE devices, compound nerve action potentials (CNAP) evoked by electrical stimulation of the medial and lateral plantar nerve (MPN and LPN), and ongoing single potentials elicited by mechanical stimuli on different areas of the paw of the animal were recorded from each electrode pole in the device (**Figure** **6**A). First, it is worth noting that not all the poles of the devices tested allowed recording of neural signals, either because they were not working, or because they were not at the adequate point in the nerve to detect the small neural signal.

**Figure 7:**
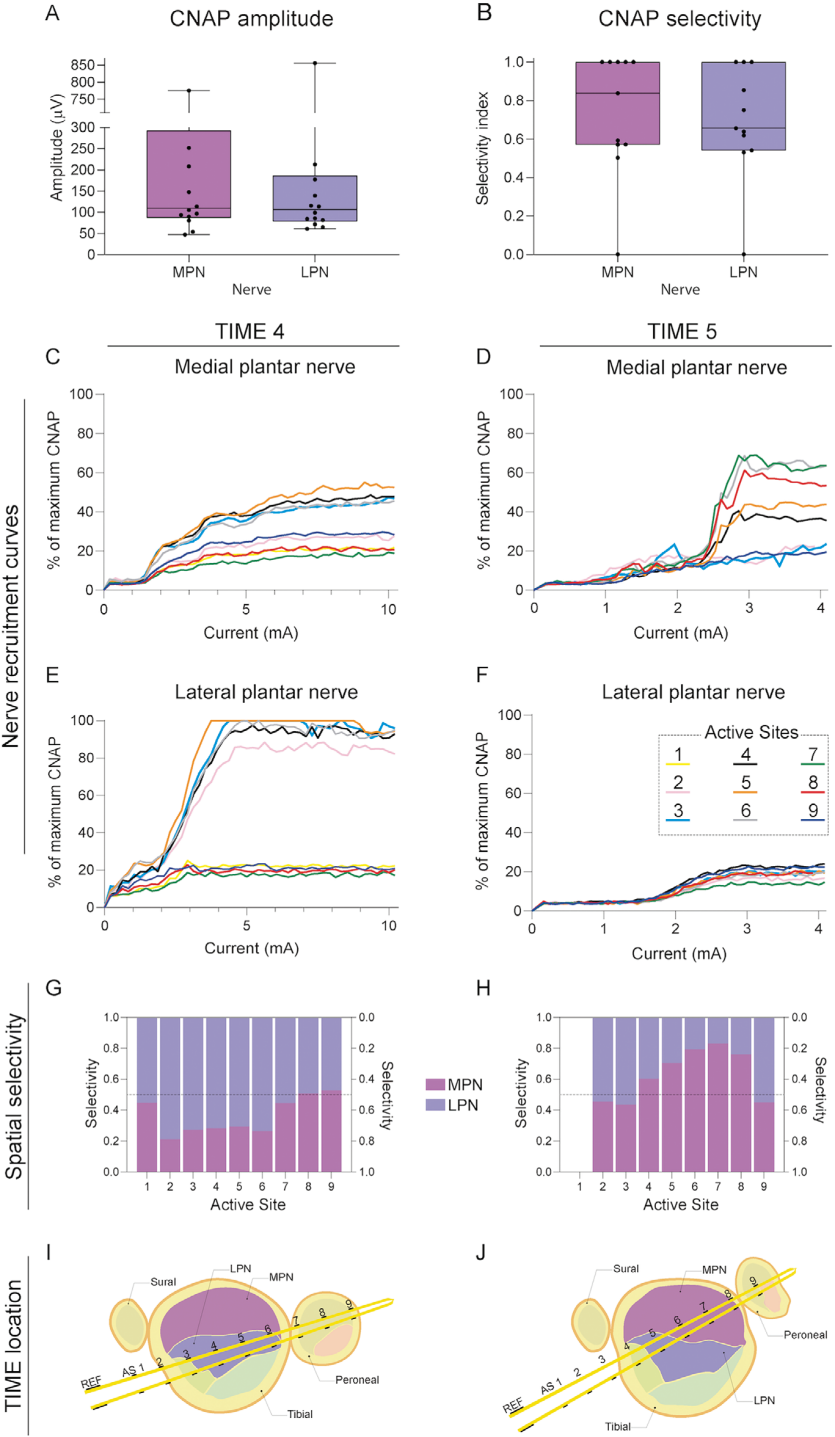
Recording capabilities of TIME EGNITE devices to electrical stimulation. A) Plots of the maximal CNAP amplitude elicited by electrical stimulation of the MPN or the LPN, recorded with the implanted devices at 0 dpi; n = 14, *p*>0.05 Mann–Whitney test. Data are represented as boxplot (box: median and interquartile range; whiskers: max and min). B) Selectivity index of the MPN and LPN CNAPs. The maximum selectivity value among all electrode poles in each device for both nerves was used for the analysis; n = 11, Mann–Whitney U test. C,F) Recruitment curves of the CNAPS elicited by stimulation of the MPN (C,D) and LPN (E,F) recorded from each pole of the TIME 4 and 5 devices placed in the sciatic nerve at 0 days. G,H) Selectivity from each electrode pole (active site) of the TIME 4 (G) and TIME 5 (H), calculated in the same step of the current increasing stimulation protocol (5 mA for the TIME 4 and 3, 12 mA for the TIME 5). I, J) The hypothetical location within the nerve of TIME 4 (I) and TIME 5 (J) devices, deduced based on selectivity data.

The EGNITE devices were able to record small CNAPs, from a few µV in amplitude, with increasing amplitude as the electrical stimulus increased in intensity (see Figure 6B). The CNAPs were recorded as triphasic potentials at a latency compatible with a conduction velocity corresponding to Aα and Aβ nerve fibers. These CNAPs were elicited by electrical stimulation of both MPN and LPN separately. All devices successfully recorded CNAPs during the acute test, with a mean maximal amplitude of about 200 µV (**Figure** **7**A), but only 2 out of 14 devices were able to do so at 30 dpi.

To assess the selectivity of CNAPs evoked from the MPN and the LPN, the SI was calculated and averaged approximately 0.7 (Figure 7B). Six devices out of 11 achieved 100% selectivity in at least one pole, meaning that it was able to record CNAPs from one nerve and not from the other nerve. Conversely, two devices obtained 0% selectivity for one of the nerves, indicating that they were unable to record the activity of one of the nerves. Finally, three devices showed a selectivity greater than 0.6 for both nerves. This suggests that in most cases (8 devices), each TIME implant was selective for interfacing one of the nerve fascicles in the implanted sciatic nerve (see schema in Figure 9J).

Two TIME EGNITE devices were used with a stimulation protocol that allowed for a higher resolution recruitment curve (more stimulation steps) (Figure 7C–F). In these two cases, only one arm of the device was analyzed due to time constraints of the in vivo procedure. The TIME 4 recorded CNAPs equal to or close to the maximum CNAP of the LPN from AS2, 3, 4, 5 and 6, while AS1, 7, 8, and 9 recorded low‐amplitude CNAPs (Figure 7E). For MPN CNAPs (Figure 7C), the amplitude of the recordings was lower, although poles that recorded high‐amplitude potentials for the LPN recorded also higher potentials for the MPN compared to the others. Plots of Figure 7G,H shows how each pole has a recording selectivity profile. In the TIME 4 (Figure 7G) AS2, 3, 4, and 5 have higher selectivity for the LPN, while AS1, 2, 7, and 8 do not show selectivity as SI values are close to 0.5. In the case of TIME 5, the recruitment curves show that AS4, 5, 6, 7, and 8 recorded higher action potentials for the MPN (Figure 7D) compared to the LPN (Figure 7F). Consequently, these poles or ASs have higher selectivity, while AS2, 3, and 9 show selectivity close to 0.5 (Figure 7H).

The analysis of the recruitment curves and selectivity indicates that the distribution of poles within the nerve affects the ability to record signals from one nerve fascicle or the other. In Figure 7I,J, a possible location of the electrode poles within the nerve is presented based on the results described above. In the case of TIME 4 (Figure 7I), it is assumed that the device passed through a region of the nerve where the fibers from the LPN are located, while the fibers from the MPN are located further, which explains why the amplitudes of the recorded signals from the MPN are lower but still not negligible. In the case of TIME 5 (Figure 7J), the poles recorded higher potentials for the MPN, so the implant is thought to be near the fibers coming from the MPN. Conversely, none of the poles recorded high amplitudes of the LPN, so these fibers could be on the opposite side.

The nerve fibers supplying both MPN and LPN are located in the same tibial branch of the sciatic nerve.^[^ ^25^ ^]^ As in the case of motor fibers innervating the GM and PL muscles, it was difficult to obtain high selectivity between axons placed in the same fascicle of the sciatic nerve. In addition, the small amplitude of the neural signals makes it difficult to detect the signals at low level of activation, thus being conducted by a few, well localized, nerve fibers. For these reasons, the selective targeting of specific sensory afferences is more difficult to achieve compared to motor efferent fibers in the study protocols used (see also).^[^ ^26^ ^]^

Regarding the recording of functional nerve signals, we performed three different maneuvers to activate sensory fibers while recording from the electrode poles of the implanted EGNITE device. The noxious pinching stimuli elicited a reflex muscular response, so that neural signals were masked by motor unit action potentials (see Figure 6C), that are quite larger in amplitude. All poles recorded these muscular signals.

**Figure 8:**
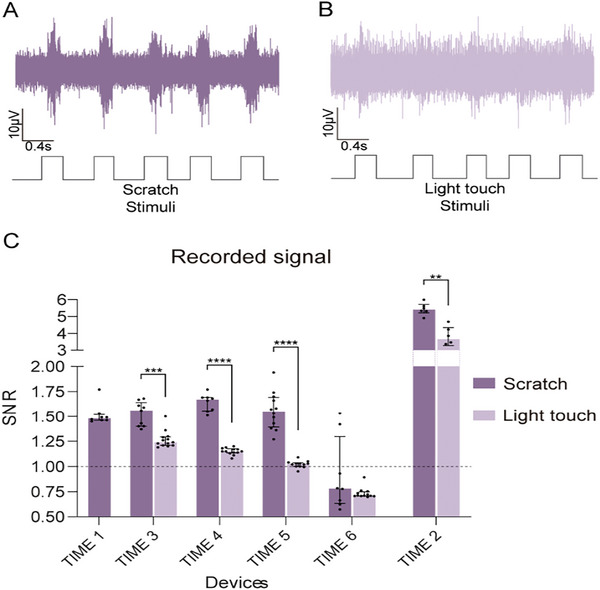
Recording capabilities of TIME EGNITE devices to mechanical stimulation. A,B) Representative raw ENG recordings elicited by mechanical stimuli on the paw by fast scratching (A), and light contact, recorded in the sciatic nerve with TIME devices. C) SNR of the mechanically elicited nerve signals in six TIME devices. In TIME 2, five poles were shunted. When the values are below the dotted line the neural signal is indistinguishable from the noise. Dots represent technical replicates. TIME 1 Scratch (S) n = 8, Light touch (LT); TIME 3 S n = 9, LT n = 13; TIME 4 S n = 8, LT n = 13; TIME 5 S n = 12, LT n = 12; TIME 6 S n = 10, LT n = 12; TIME 2 S n = 6, LT n = 6. ^**^ *p* < 0.01, ^***^ *p* < 0.001, ^****^ *p* < 0.0001, Mann–Whitney test. Data are represented as median and interquartile range.

Fast and slight scratch of the sole of the paw represents a dynamic stimulus that activates numerous mechanoreceptors of the sole. It was easy to detect the signals from the background noise, with an average SNR close to 1.5 (**Figure** **8**A,C). Pressure on small skin areas with the Von Frey filament activated fewer and more localized mechanoreceptors, thus, it was more difficult to differentiate action potentials from the noise (Figure 8B,C), with a lower SNR. The SNR values calculated for the mechanical stimuli are similar to those obtained by Badia et al.^[^ ^26^ ^]^ using TIME devices containing larger IrOx electrodes. To improve the recording capabilities of EGNITE devices we compared recording using 5 poles joined, so the recording area was larger. With this configuration nerve signals were easier to distinguish from the noise (Figure 8C, TIME2). Moreover, it was possible to record signals elicited by pressing specific areas of the paw more often than with single poles.

## 3 Discussion

The results of the present study indicate that the newly generated engineered graphene, EGNITE, is safe for in vivo implantation, and that intraneural electrodes with EGNITE as conducive material allow for effective nerve stimulation, with comparatively low current needed, and for high resolution signal recording. As recently reported in detail,^[^ ^17^ ^]^ the graphene‐based EGNITE electrodes used can be miniaturized to the microscale and integrated in flexible, thin‐film fabrication processes, particularly on polyimide substrate in designs adequate for neural applications,^[^ ^2^ ^]^ while keeping their properties for bidirectional neural interfacing. EGNITE advantages are based on its highly porous structure and a reversible charge injection mechanism, able to sustain long‐term stimulation with contacts of small size.

### 3.1 Biocompatibility of the EGNITE Electrodes

The biocompatibility of different forms of graphene that could be used for neural interfaces has previously been tested in vitro,^[^ ^27^, ^28^, ^29^, ^30^, ^31^, ^32^ ^]^ but only in a few in vivo experiments of macroscopic devices^[^ ^33^ ^]^ and even fewer in nervous tissue.^[^ ^34^, ^35^ ^]^ Moreover, significant changes are made in the structure of graphene to further increase its performance, that may also alter its biocompatibility and stability^[^ ^36^ ^]^ and even induce toxicity. The graphene material used in this work was newly developed from reduced oxide graphene to create EGNITE,^[^ ^17^ ^]^ thus, a biocompatibility assay was performed first. In the in vivo study, the devices used contained much larger amount of EGNITE than necessary for functional electrodes; in the case of a LIFE, about 16–20 electrodes of 25 µm diameter, whereas the devices used contained about 20 times more dots. Besides, the biocompatibility was tested with mock non‐functional devices without connecting wires, that may induce some tethering forces in the nerve,^[^ ^2^ ^]^ avoiding external artifacts in the progression of the FBR, whereas focusing only on the reaction to the implanted material.

Cytotoxicity evaluations made with primary DRG cells and primary cortical cells seeded on the PI substrate alone and containing EGNITE showed that both components were inert, and indeed, neuronal cells grew and readily extended neurites on top of the EGNITE coated areas. In the in vivo evaluation, devices containing a large amount of EGNITE did not induce neural damage, and the results were indistinguishable from those of the PI alone, and similar to those of sham operated animals. The present results with PI+EGNITE are in agreement with similar studies of intraneural electrodes made of PI plus Pt or IrOx,^[^ ^12^, ^20^, ^37^ ^]^ in which slight functional decline was observed at early days but recovered over a few weeks. This evolution suggests that the surgical implantation procedure is the main reason for these variations and causes only a mild and temporary functional deficit without evidence of axonal damage.

The histological analysis of the FBR was aimed to characterize three specific time points of the process, the inflammation peak occurring at 2 weeks and the late stabilization at 8 and 12 weeks after the implantation, as characterized in similar implants performed in our laboratory.^[^ ^22^ ^]^ During the early phase of FBR, resident macrophages are stimulated by the damage induced by device implantation and protein adsorption to the biomaterial. Systemic macrophages are also recruited by chemoattractant factors to the damaged area. This early inflammatory phase occurs during the first two weeks. From this time on, the number of macrophages decreases, and a stabilization phase occurs in which invasion of fibroblasts predominates. Since medical implants are generally too large to be fully degraded, fibroblasts around the implant generate a connective capsule that isolates it from the body. The thicker the capsule, the more difficult the interaction of the neural electrode with the tissue is. The FBR of the PI+EGNITE implanted devices showed a similar evolution to that of PI alone devices, as well as to that previously described in other PI devices,^[^ ^20^, ^38^ ^]^ both in terms of inflammatory infiltration and connective capsule around the electrode device.

### 3.2 Functionality of the EGNITE Electrodes

Neural stimulation delivered through the TIME EGNITE electrodes was proven to activate specific subsets of axons within the fascicles of the sciatic nerve with low current thresholds in acute and sub‐chronic conditions. Neural recording of CNAP and ENG elicited by mechanical stimuli were feasible but were more dependent of the decreased functionality over time.

Metal electrodes including stainless steel, tungsten, platinum, platinum–iridium alloys, IrOx, and titanium nitride have been used due to its electrical capabilities for effective stimulation and recording of the nervous system.^[^ ^39^ ^]^ To provide complex exchange of signals, as needed for natural‐like sensations and accurate control of the movement of a prosthesis, it is mandatory to stimulate and record small populations of afferent and efferent axons in a peripheral nerve reliably and selectively.^[^ ^40^ ^]^ Thus, to increase selectivity and spatial resolution, the size of the active electrodes must be reduced while increasing the number of them interfacing the nerve. When the size of metallic electrodes is reduced to a micrometer scale, the threshold current to produce the stimulation of the nervous tissue is generally above the charge injection limit (CIL) characteristic of the metal. Exceeding the CIL of the material leads to faradic reactions in electrode‐tissue interface that compromise biocompatibility and integrity of the conductive material, reducing long‐term stability. Likewise, the reduction of electrode surface area increases interfacial impedance of the electrode, which translates into recording with lower SNR.^[^ ^39^ ^]^ Besides, a critical challenge lies in maintaining functional neuroprostheses over extended periods of time that requires stability and robustness of the interface device. New materials have been explored to improve classic metallic electrodes, for example, conductive polymers such as PEDOT or materials derived from carbon.^[^ ^9^ ^]^ Among carbon materials, graphene and graphene‐derived materials have stood out for their electrical and electrochemical performance and their suitability for integration into flexible devices.^[^ ^14^, ^17^ ^]^

#### 3.2.1 Nerve Stimulation

Given the improvement in the electrical performance of EGNITE compared to other materials,^[^ ^17^ ^]^ it has been possible to reduce the size and therefore increase the number of electrode contacts that fit into a TIME designed for the rat sciatic nerve. In previous studies with similar TIME design on PI, five poles of 60 µm in diameter made out of platinum (Pt; 300 nm in thickness) per arm,^[^ ^41^ ^]^ and four poles of IrOx contacts with a diameter of 80 µm had been used.^[^ ^23^, ^24^ ^]^ Cutrone and colleagues, used the intraneural electrode SELINE, with 5 poles per arm of gold whose area was 3700 µm^2^ each.^[^ ^19^, ^42^ ^]^ In comparison, our EGNITE based device has 9 electrodes of 25 µm in diameter per arm, of which at least 7–8 are always inside the nerve. Moreover, the nerve activation threshold current was two to three times less compared to those found in these previous studies,^[^ ^23^, ^41^, ^42^ ^]^ which may lower power consumption in future long‐lasting implantable neuroprostheses, representing an important advantage of EGNITE as active element.

Regarding selectivity for stimulation of small nerve fascicles, in the model used the SI for activation of the three tested muscles ranged between 0.6 and 0.9, similar to values found in previous studies using TIME design of electrodes.^[^ ^23^, ^24^ ^]^ It is worth noting that the transversal insertion of the TIME allows for enhanced selectivity when compared to cuff extraneural or longitudinal intrafascicular electrode designs.^[^ ^26^, ^41^ ^]^ Interfascicular selectivity was easy to achieve because of the separation by the perineurium of the tibial and peroneal fascicles, as it was shown by the high SI of the TA muscle, innervated by axons running in the peroneal fascicle. On the contrary, intrafascicular selectivity was more difficult for muscles innervated by axons in the same tibial fascicle, thus closer and without a perineurial barrier, as indicated by the lower SI of the GM muscle. It was expected that smaller electrodes would improve the focalization of stimuli to the target fibers. However, we did not find higher selectivity than with larger metal electrodes used in the cited previous studies. There are two plausible reasons. First, the TIME design by itself allows a high selectivity of stimulation in the peripheral nerve.^[^ ^41^ ^]^ Second, the small size of the rat sciatic nerve used in the study, in which the localization of small subfascicles innervating different muscles in the limb is quite close,^[^ ^25^ ^]^ limits the possibilities to selectively activate them when the injected current spills over small distance from the pole.

#### 3.2.2 Nerve Recording

Recording nerve signals is a complicated task compared to recording muscle signals. This difficulty mainly arises because, due to the small size of axons, extracellularly recorded signals are in the microvolt range, as opposed to muscle signals in the millivolt range. The noise present to a greater or lesser extent in all recording systems can hinder low‐amplitude signal acquisition. Moreover, the electrode size affects its ability to record signals primarily due to increased electrode impedance and thermal noise, which impact the bandwidth of the electrical recordings.^[^ ^43^ ^]^

The EGNITE based electrodes were able to record CNAPs of a similar amplitude to those recorded with cuff electrodes,^[^ ^44^ ^]^ or with other TIME implanted within the rat sciatic nerve.^[^ ^23^ ^]^ Additionally, the capacity to selectively record CNAPs from two tributaries of the sciatic nerve, the MPN and the LPN, which innervate the medial and lateral parts of the rat paw, was investigated. Most devices (8 out of 11) were selective for only one nerve. This fact can be explained by the wide area occupied by the axons coming from each plantar nerve,^[^ ^25^ ^]^ so it may be possible that the TIME crosses the tibial nerve though one or the other. The remaining devices tested were able to record the CNAP of the MPN and LPN with moderate selectivity. These results agree with those found by Badia et al.^[^ ^26^ ^]^ As in the case of stimulation, selectively recording nerve signals in the same fascicle is complicated because there is no epineurium that isolates nerve signals from different axonal populations.

The EGNITE device was also able to record single neural potentials elicited by mechanical stimuli applied to the animal paw. Motor unit action potentials from nearby muscles were easily recorded during withdrawal response to painful stimuli. Despite the small size of the electrodes (25 µm diameter), signal recording was possible with a SNR similar to other devices with larger ones.^[^ ^23^, ^26^ ^]^

### 3.3 Advantages, Limitations, and Further Work

A key advantage of the EGNITE material is its high charge injection capabilities (2–5 mC cm^−2^),^[^ ^17^ ^]^ much higher than in typically used noble metal electrode materials. This high charge injection capacity originates from EGNITE nanoporous structure and large electrochemically usable potential window; and allows electrode miniaturization to smaller areas than metal‐based electrodes while preserving nerve stimulation capabilities. This concept was proved by our results, and led to other advantages, such as a significantly smaller current needed to activate the nerve fibers with the EGNITE electrodes compared to noble metal electrodes of larger size.

One aspect of stimulation and recording that has not been addressed in this study is the electrical configuration of the electrode and the parameters of the stimulation protocol. The monopolar configuration, using a common reference electrode within the device, has been used in this work. However, it has been demonstrated that bipolar, tripolar, or multipolar configurations may have a positive impact on the functionality of electrodes in both stimulation,^[^ ^41^, ^45^, ^46^ ^]^ and recording modes.^[^ ^47^, ^48^, ^49^ ^]^ For stimulation, the multipolar configuration aims to actively restrict the field of excitation, consequently increasing selectivity. The selectivity of stimulation may be also affected by the stimulus pulse characteristics. Specifically, it has been shown that short pulses (20 µs) and the introduction of a hyperpolarizing pre‐pulse may improve stimulation selectivity.^[^ ^50^ ^]^ Regarding neural activity recordings, the multipolar configuration in cuff devices improves the spatial selectivity of the recorded tissue due to the higher number of AS. Moreover, the noise decrease improves the quality of signal recordings.^[^ ^47^, ^49^, ^51^ ^]^ Therefore, future studies using the newly developed EGNITE‐based electrodes may assess refinements in the electrode configurations to improve selectivity and efficiency of the bidirectional communication.

In addition to the electrode electrical properties, characterization of the anatomical and functional integration in the nervous tissue and of the FBR to the implanted device, are necessary steps for improving the clinical use of neural interfaces.^[^ ^39^ ^]^ The stability in position of the electrode AS inside the nerve along time is compromised due to the movements of the limbs, and it will be advantageous to add some anchoring mechanism to the device substrate, as attempted in the SELINE design,^[^ ^42^ ^]^ but avoiding potential structural damage. Better integration may be achieved also by using more flexible materials as the electrode substrate.^[^ ^52^ ^]^ The stimulation threshold charge and the impedance of neural electrodes progressively increase during the first month post‐implantation and thereafter tend to stabilize, as found in this work and in previous studies.^[^ ^19^, ^23^, ^53^ ^]^ This is in part due to the FBR generated from the electrode implantation, that results in the formation of a fibrous capsule around the device that progressively separates axons away from the electrodes contacts and increases the impedance. Thus, to improve the long‐term functionality of intraneural electrodes, different strategies have been developed, including surface coatings with biomimetic hydrogels, local release or systemic administration of anti‐inflammatory drugs.^[^ ^54^ ^]^

Therefore, we expect high‐market competitiveness of our EGNITE material, particularly in applications where localized high‐charge injection is advantageous, such as selective nerve activation for neuroprosthetics. Indeed, progress in materials science has enabled the creation of biocompatible, neuroimplantable devices, employing flexible substrates and novel form factors, further resulting in development of devices approved for medical use.^[^ ^55^ ^]^ Based on the reported results, we consider that EGNITE‐based microelectrode arrays have high‐potential for becoming part of the next generation interfaces in neuroprosthetic applications such as brain, retinal, extraneural or intraneural implants for sensory and motor recovery,^[^ ^56^ ^]^ as well as for bioelectronic therapeutic approaches being explored in inflammatory and autoimmune diseases, diabetes or cardiovascular diseases.^[^ ^57^ ^]^

## 4 Experimental Section

### In Vitro Biocompatibility Study

**Figure 9:**
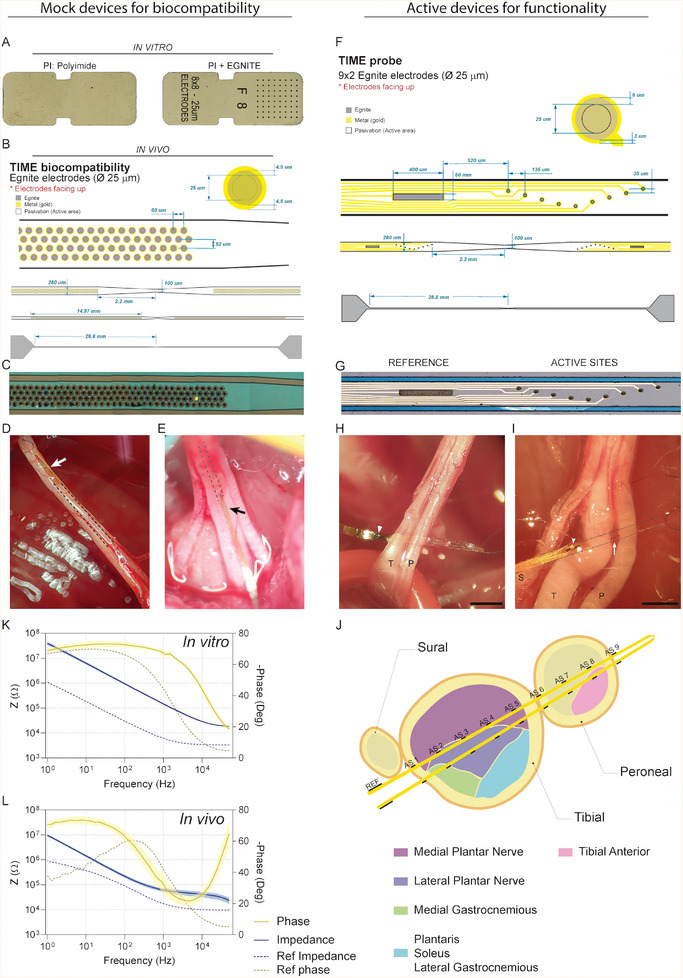
Implants design. A) Substrates for in vitro biocompatibility tests made of PI or PI+EGNITE. Black dots are made of EGNITE material (64 black dots, ø 25 µm). B) Probe design for in vivo biocompatibility tests. C) Image of a section of a fabricated probe with many EGNITE microelectrodes (black dots) D) Microphotograph of a sciatic nerve and a longitudinally implanted PI device already inside the nerve. The arrow indicates the tip of the implant outside the nerve. E) Detail of a device with EGNITE inside a sciatic nerve. The arrow indicates the insertion point, and the dashed line indicates the placement of the intraneural device within the tibial fascicle. Scale bar: 1 mm. F,G) Image of a section of a fabricated probe for in vivo functional tests with 9 EGNITE microelectrodes (black dots) plus a reference (black rectangle). H,I) Detail of a device within the sciatic nerve. White arrowheads indicate the reference electrode. The white arrow indicates some ASs or microelectrodes between the tibial and peroneal fascicles of the sciatic nerve. S: Sural, T: Tibial, P: Peroneal. Scale bar: 1 mm. J) Schematic cross section of an implanted TIME with electrodes, named active sites (AS 1–9) crossing the tibial and peroneal fascicles. Subfascicle topography of the rat sciatic nerve is extracted from Badia et al. (K,L).^[^ ^25^ ^]^ Electrochemical impedance spectroscopy of EGNITE AS and reference electrode showing the module of the impedance and the phase versus frequency in vitro K) and in vivo L) (n  =  9 AS). Data are represented as mean and 95% confidence intervals (color‐shaded area).

DRG neurons or cortical neurons from Sprague–Dawley (SD) rats were cultured on top of three different substrates: control culture glass, PI as the substrate of the neural device, and the PI substrate containing EGNITE (**Figure** **9**A). The pieces containing PI and PI+EGNITE were glued to the culture glass with collagen. Neuron viability after 4 or 7 days in culture was assessed by the MTT (3‐(4,5‐dimethylthiazol‐2‐yl)‐2,5‐diphenyltetrazolium bromide) cell viability test and after immunohistochemical labeling. Both primary cell cultures were performed 3 times with 4 replicates of each substrate condition.

*DRG Culture*: Rats 21 days old were euthanized with pentobarbital. DRG were extracted and kept in cold Gey's balanced solution with 2% glucose. Cleaned ganglia underwent enzymatic digestion with trypsin 1x, collagenase 1x and DNase (1 mg mL^−1^) diluted in Hank's Balanced Salt Solution (HBSS, Gibco) for 30 min at 37 °C, followed by mechanical digestion. Cells were then filtered with a 70 µm sterile filter to remove myelin fragments and centrifuged at 900 rpm for 7 min. Neurons were counted in a Neubauer chamber after homogenization. Four wells per condition were seeded with a concentration of 8000 cells ml^−1^ in 24 multiwell plates (500 µL per well) pretreated with poly‐D‐lysine (0.01 mg ml^−1^) and laminin (1 mg ml^−1^). Cells were maintained in Neurobasal A medium enriched with 2% B27, 2% glucose, 1% glutamine, and 1% penicillin and streptomycin. The medium was changed after 1 and 3 days of culture.

*Cortical Cells Culture*: E17 rat embryos were used for the culture of cortical cells. Briefly, female pregnant rats were euthanized with pentobarbital, the embryos were extracted and the cerebral cortex dissected. The meninges were removed and cleaned. Cortices were kept in Krebs–Ringer Buffer solution with trypsin and DNase for enzymatic digestion for 10 min at 37 °C, followed by mechanical digestion. Cells were then filtered with a 70 µm sterile filter, centrifuged at 1000 rpm for 5 min and neurons were counted in a Neubauer chamber after homogenization in DMEM containing 10% FBS. Four wells per condition were seeded with a concentration of 250000 cells ml^−1^ in 24 multiwell plates (500 µL per well) precoated with poly‐D‐lysine (0.01 mg ml^−1^). Cells were seeded in DMEM medium enriched with 2% B27, 2% glucose, 1% glutamine and 1% penicillin and streptomycin. After 24 h the medium was replaced with Neurobasal medium enriched with 2% B27, 2% glucose, 1% glutamine and 1% penicillin and streptomycin, and replaced every 3 days.

*Viability Evaluation and Immunolabeling*: At 4 and 7 days in vitro for DRG and cortical cells respectively, an MTT assay was performed to determine cell viability (n = 3). For this purpose, culture medium was replaced with medium containing 0.15 mg ml^−1^ MTT, maintained for 1 h, and cells were lysed with DMSO. Absorbance was read out through a spectrophotometer (Bio‐tek) at 560 nm wavelength and data collected using KC Junior software. Readings were normalized against the control group, in which cells grew on coverslips, to obtain the percentage of cell survival.

For immunofluorescence labeling, coverslips containing cells were fixed for 20 min with paraformaldehyde (PFA). After blocking with normal donkey serum, slides were incubated with primary mouse antibody against β3 tubulin (1:500; Biolegend) overnight at 4 °C. Cells were then washed with 0.1% Tween buffer solution and incubated with AlexaFluor 488 donkey anti‐mouse secondary antibody (Invitrogen) for 1 h at room temperature. Finally, coverslips with cells were mounted with Fluoromount (Sigma). Images were taken with an epifluorescence microscope (Eclipse Ni, Nikon) and a digital camera (DS‐Ri2, Nikon).

### In Vivo Biocompatibility Study

To assess the biocompatibility of the developed EGNITE material, PI devices coated or not with EGNITE were longitudinally implanted in the sciatic nerve of rats for 2, 8, or 12 weeks. The intraneural test device was designed as a longitudinal strip in which the area of EGNITE in contact with the nerve was increased by a factor of 20 with respect to conventional EGNITE neural interface devices (Figure 9B,C), aiming to maximize the contact area of the new material with the tissue and to investigate immune responses.

All the in vivo experimental procedures performed complied with the ARRIVE guidelines and were approved by the Ethics Committee of the UAB in accordance with the European Communities Council Directive 2010/63/EU. Adequate measures were taken to minimize pain and animal discomfort during surgery and in the postoperative follow‐up.

Operations were performed under anesthesia with ketamine/xylazine (90/10 mg k^−1^g i.p.) on 26 female SD rats weighing 300–350 g. The sciatic nerve was surgically exposed at the midthigh and carefully freed from adherences to surrounding tissues. PI (PI‐2611, HD MicroSystems) devices with no EGNITE (used as controls) and PI devices with EGNITE were inserted longitudinally into the tibial branch of the sciatic nerve with the help of a straight needle attached to a 10–0 loop thread (STC‐6, Ethicon), as designed for longitudinal intrafascicular electrodes (LIFE) (Figure 9D,E).^[^ ^12^, ^22^ ^]^ Insertion was monitored under a dissection microscope to ensure the correct placement of the device. The wound was sutured in plane and disinfected with povidone iodine. After surgery, all animals were housed under standard conditions. The incision wounds healed without inflammatory signs and no postoperative complications were observed.

*Electrophysiological and Functional Evaluation*: Electrical performance of EGNITE microelectrodes was assessed in vitro and in vivo using electrochemical impedance spectroscopy (modulus and phase) with a potentiostat (PalmSense 4) in a frequency range between 1 Hz and 50 KHz (Figure 9K,L). The obtained values were in the same range of other metal‐based intraneural electrodes.^[^ ^13^, ^42^ ^]^

The functional properties of the nerves that had been implanted were evaluated by means of nerve conduction, algesimetry and locomotion tests at 2, 8, and 12 weeks after the implantation. The nerve conduction test was performed by stimulating the sciatic nerve proximally with single electrical pulses and recording the CMAP of the TA, GM, PL muscles as previously described.^[^ ^22^ ^]^ The nociceptive threshold to mechanical stimuli was evaluated using an electronic Von Frey algesimeter (Bioseb, Chaville, France).^[^ ^58^ ^]^ Rats were placed on a wire net platform in plastic chambers, and a metal tip was applied to the sole of the hind paw until the rat withdrew the paw in response to the stimulus. The walking track test was performed to assess locomotor function after the implant. The plantar surface of the hind paws was painted with blue ink and the rat was left to walk along a corridor. The print length, the distance between the 1st and 5th toes and between the 2nd and 4th toes were measured to calculate the SFI.^[^ ^59^ ^]^

*Histological Evaluation*: After 2, 8, or 12 weeks, animals were deeply anesthetized with an overdose of pentobarbital and transcardially perfused with 4% PFA in phosphate buffer (PB). The sciatic nerve including the implant was harvested, post‐fixed in 4% PFA in PBS for 1 h and stored in 30% sucrose in PB for cryoprotection.

Analysis of infiltrating macrophages and capsule thickness in the implanted nerves was performed by immunohistochemistry. The nerve segment containing the device implanted was sliced (15 µm thick sections) with a cryostat (Leica CM190). After thawing and blocking with normal donkey serum, slides were incubated with primary antibodies rabbit against Iba1 (1:500; Wako) for macrophages, CD90 (1:150; BD Pharmingen) for fibroblasts, and RT97 (1:200; Developmental Studies Hybridoma Bank) for axons overnight at 4°C. Slides were then washed with a 0.1% Tween 20 in PBS solution and incubated with AlexaFluor 488 donkey anti‐mouse and AlexaFluor 555 donkey anti‐rabbit secondary antibodies (Invitrogen) for 1 h at room temperature. Finally, slides were mounted with Mowiol containing DAPI (Sigma). The number of Iba1 positive macrophages in the whole tibial nerve cross section was quantified using a macro routine for Image J software. The capsule thickness was analyzed by dividing the area of the capsule by the length of the implant in the transversal section. The area was quantified as the non‐labeled space between the implant and the first axons labeled with RT97.^[^ ^24^ ^]^ As each implant has two arms, the capsule thickness of an implant is the mean of both arms. Images were taken with an epifluorescence microscope (Eclipse Ni, Nikon) and a digital camera (DS‐Ri2, Nikon). To determine the amount of collagen induced by the FBR around the implant, other cryostat sections were processed with Masson's trichrome stain.

### In Vivo Functionality Study

*Electrode Design*: Microelectrode arrays for the in vivo functionality study were designed for intraneural implantation following the TIME design.^[^ ^13^, ^41^ ^]^ The device consisted of two linear arrays of 9 circular microelectrodes (ø 25 µm) and a reference electrode (0.02 mm^2^) along a 1.2 mm strip (Figure 9F,G). Each linear array is placed at opposite sides of the stripe. EGNITE microelectrodes are made of a thin film of hydrothermally reduced graphene oxide stacked on top of gold. Arrays of EGNITE microelectrodes were integrated into flexible devices (total thickness of 13 µm) using biocompatible PI as substrate and insulation, and gold for the tracks. The PI strip has a total length of 57.6 mm and a width of 280 µm. At the center of the strip, it is narrowed down to only 100 µm. For implantation, the strip is folded at the midline to align the left and right sides of the strip and to create an arrow‐like shape at the tip of the device, enabling penetration into the nerve. The PI strip is widened at the ends (contacts pad area) to connect the device with the external equipment through a ZIF multiconnector.^[^ ^17^ ^]^

*Nerve Implantation*: To assess the functionality of the TIME EGNITE devices, they were transversally implanted in the sciatic nerve of rats. After surgery, all animals were housed under standard conditions. Electrophysiological studies of electrical stimulation and recording using the implanted devices were performed acutely and at 30‐ and 60‐dpi.

Operations were performed under anesthesia with ketamine/xylazine (90/10 mg k^−1^g i.p.) on female SD rats. The sciatic nerve was surgically exposed at the mid‐thigh and freed from adherences to surrounding tissues. Nerves and EGNITE devices were delicately handled with fine Dumont forceps. The devices were inserted transversally across the tibial and peroneal branches of the sciatic nerve (Figure 9J) with the help of a straight needle attached to a 10–0 loop thread (STC‐6, Ethicon)^[^ ^20^, ^41^ ^]^ the thread was passed between the two arms of the device and pulled the arrow‐shaped center of the electrode strip. The insertion was monitored under a dissection microscope to ensure correct placement of the device (Figure 9H,I).

After conducting nerve stimulation and recording protocols, the device was attached to adjacent muscle tissue using two suture stitches. In addition, fibrin glue or KwikKast were used to keep the implanted electrode in place during the time of implantation. To easily access the electrode contacts in chronic experiments, the pads portion of the devices was passed through the muscular incision and placed subcutaneously at the side. The pads were protected with a plastic envelope sealed with KwikKast. The plastic envelope was placed under the skin, the muscle incision was closed with stitches and the skin wound was closed with staples.

*Nerve Stimulation Protocol*: The stimulation protocol is summarized in Figure 4. To assess the stimulation performance of the implanted electrodes (n = 19), electrical stimulation was applied with the EGNITE devices to the sciatic nerve. Biphasic current pulses (pulse width of 100 µs) were delivered through each one of the electrodes (or poles) against a common reference within the device ribbon part or a needle reference electrode placed near the sciatic nerve. Current pulses with increasing intensity were delivered by a Digitimer DS4 stimulator.

CMAPs were recorded from TA, GM, and PL muscles using small needle electrodes placed in each muscle belly.^[^ ^24^ ^]^ The CMAPs were amplified (P511AC, Grass), band‐pass filtered (3 Hz–3 kHz), and digitized with a Powerlab system (PowerLab16SP, ADInstruments) at 20 kHz using LabChart software. The amplitude of each CMAP was measured peak to peak and normalized to the maximum CMAP amplitude obtained in each experiment by stimulation of the sciatic nerve with two needle electrodes. For each electrode, the threshold current of stimulation which elicited 5, 30, and 95% of the maximum CMAP was determined. The electrode pole with the lowest threshold value in each device was used for analysis.

Finally, the SI was calculated (Equation 1) to quantify the specific activation of a single muscle among the set of three muscles (GM, PL, TA), as previously described.^[^ ^41^, ^45^ ^]^ The selectivity was considered for recorded CMAPs/CNAPs of 5% and 30% of the maximum CMAP/CNAP.

(1) $$\mathrm{SI}=\frac{CMAP}{\sum_{j}CMAP_{\mathrm{nj}}}$$

(2) $$\mathrm{SI}=\frac{CNAP}{\sum_{j}CNAP_{\mathrm{nj}}}$$

*Nerve Recording Protocol*: To assess the recording capabilities of EGNITE electrodes, two different protocols were performed, previously described by Badia et al. (see Figure 6A).^[^ ^26^ ^]^

First, CNAPs were recorded from each one of the electrode poles following electrical stimulation of the distal MPN and LPN at the hind paw (n = 14). Ten monophasic rectangular pulses of 10 µs from 1 to 10 mA intensity (Grass S44 with PSIU6 stimulus isolation unit) were delivered using two small needles inserted on the medial or lateral side of the paw. The amplitude of each CNAP was measured peak to peak and normalized to the maximum CNAP amplitude recorded in each experiment with hook electrodes around the sciatic nerve. The SI was calculated (n = 11) to quantify the specific activation of a nerve among the set of two nerves (Equation 2). For selectivity calculations, recorded nerve potentials were considered if they were above 5% of the maximum CNAP. In two additional devices (TIME 4 and TIME 5) a slightly different protocol was used to produce a smoother recruitment curve; 50 biphasic rectangular pulses of 100 µs and up to 10 mA (DS4 Stimulator, Digitimer) were delivered.

For the second protocol, sensory activity was evoked by applying a thin probe to softly scratch the sole of the paw, by contacting with a Von Frey monofilament specific areas of the paw, and by pinching a toe with tweezers to elicit a noxious stimulus. ENG recordings were amplified x1000, band‐pass filtered (between 300 Hz and 10 kHz) and fed to a power‐line noise eliminator (Hum Bug, Quest Scientific), then digitized at 20 kHz and recorded with LabChart software (PowerLab System, ADInstruments). The total power of the recorded signals and the noise (no stimulus applied) was obtained after applying the short‐time Fourier transform with a window of 1 ms, and an overlap of 87.5%. The best recording pole in each TIME was used to calculate the SNR, defined as the ratio between the mean of the total power when the stimuli are applied and the mean of the total power when there are no stimuli applied.

### Statistical Analysis

The normality of the data was checked with the Shapiro–Wilk test. Statistical comparisons of normal data were made using the appropriate parametric test, either One‐way ANOVA followed by Dunnett's multiple comparison tests or Two‐ways ANOVA followed by Sidak's multiple comparison tests. Normal distributed data are presented as mean ± SEM. Statistical comparisons of non‐normal distributed data were made using appropriate non‐parametric test, two‐tailed Mann–Whitney or Kruskal–Wallis tests followed by Dunn's multiple comparison test. Non‐normal distributed data are presented as median and interquartile range. The test applied in each case and the sample size is specified in the figure captions. Electrophysiological values in the functional tests were normalized to the maximal action potential amplitude of the same animal for each test session. Differences were considered significant when p < 0.05. The GraphPad Prism 8 software was used for statistical analyses.

## Conflict of Interest

D.V. and J.A.G. hold interest in INBRAIN Neuroelectronics that has licensed the technology described in this manuscript.

## Author Contributions

X.N. and J.dV. conceived and designed the study, supervised the experiments, and revised the manuscript. B.R.M. performed experiments, analyzed the data, prepared figures, and wrote the draft manuscript. D.V., S.T.W., E.M.C., N.R., and J.A.G. developed the EGNITE material and the microelectrode technology and provided the devices for biocompatibility and functionality studies. All the authors reviewed, edited, and approved the manuscript.

## Data Availability

The data that support the findings of this study are available from the corresponding author upon reasonable request.
